# Spikes and High Frequency Oscillations in Lateral Neocortical Temporal Lobe Epilepsy: Can They Predict the Success Chance of Hippocampus-Sparing Resections?

**DOI:** 10.3389/fneur.2022.797075

**Published:** 2022-08-01

**Authors:** Alessandra Maccabeo, Maryse A. van 't Klooster, Eline Schaft, Matteo Demuru, Willemiek Zweiphenning, Peter Gosselaar, Tineke Gebbink, Wim M. Otte, Maeike Zijlmans

**Affiliations:** ^1^Department of Neurology and Neurosurgery, University Medical Center Utrecht Brain Center, University Medical Center Utrecht, Utrecht, Netherlands; ^2^Stichting Epilepsie Instellingen Nederland, Heemstede, Netherlands

**Keywords:** epilepsy surgery, seizure outcome, temporal lobe epilepsy, intraoperative electrocorticography (EcoG), mesiotemporal areas

## Abstract

**Purpose:**

We investigated the distribution of spikes and HFOs recorded during intraoperative electrocorticography (ioECoG) and tried to elaborate a predictive model for postsurgical outcomes of patients with lateral neocortical temporal lobe epilepsy (TLE) whose mesiotemporal structures are left *in situ*.

**Methods:**

We selected patients with temporal lateral neocortical epilepsy focus who underwent ioECoG-tailored resections without amygdalo–hippocampectomies. We visually marked spikes, ripples (80–250 Hz), and fast ripples (FRs; 250–500 Hz) on neocortical and mesiotemporal channels before and after resections. We looked for differences in event rates and resection ratios between good (Engel 1A) and poor outcome groups and performed logistic regression analysis to identify outcome predictors.

**Results:**

Fourteen out of 24 included patients had a good outcome. The poor-outcome patients showed higher rates of ripples on neocortical channels distant from the resection in pre- and post-ioECoG than people with good outcomes (*p*_*pre*_ = 0.04, *p*_*post*_ = 0.05). Post-ioECoG FRs were found only in poor-outcome patients (*N* = 3). A prediction model based on regression analysis showed low rates of mesiotemporal post-ioECoG ripples (OR_*mesio*_ = 0.13, *p*_*mesio*_ = 0.04) and older age at epilepsy onset (OR = 1.76, *p* = 0.04) to be predictors of good seizure outcome.

**Conclusion:**

HFOs in ioECoG may help to inform the neurosurgeon of the hippocampus-sparing resection success chance in patients with lateral neocortical TLE.

## Introduction

Temporal lobe epilepsy (TLE) is the most common cause of drug-resistant epilepsy, and the success chance of seizure freedom after TLE surgery is around 60–70% (1, 2).

Intraoperative electrocorticography (ioECoG) is used to tailor resection based on interictal epileptiform discharges, e.g., spikes, in dedicated surgical centers. The presence of spikes before resection (pre-ioECoG) can guide the neurosurgeon, and after the resection residual events may push to extend it, although the effectiveness of removing cortex showing spikes observed during ioECoG has been debated (3–8).

Temporal lobe epilepsy is considered a network disease, (9) and the temporal neocortical lesions can generate secondary epileptogenicity in mesiotemporal structures (10, 11). The hippocampus in neocortical TLE frequently shows spikes even if not involved in the lesion, (11, 12) and the presence of residual spikes recorded from the hippocampus seems to predict poor seizure outcome (13). Although complete removal of the hippocampus and amygdala improves seizure outcome after surgery, it can negatively affect cognitive function and memory (2, 14).

High-frequency oscillations (HFOs; divided in ripples, 80–250 Hz, and fast ripples, 250–500 Hz) are proposed as a potential new biomarker of epileptogenic tissue in the ioECoG.

High-frequency oscillations seem to be more specific markers for epileptogenicity in comparison to spikes (15, 16). Several retrospective studies showed that resection of HFO-generating areas is associated with good seizure outcome, (16–19) whereas other studies found no such correlation or differences among centers (20–23). Recently, it was shown that residual FRs in the post-ioECoG, especially given the presence of pre-resection FRs, are predictors of seizure recurrence (24–26).

Ripples do not show this predictiveness, and this could be explained by the fact that physiological ripples exist too. They have been found in the eloquent cortex, e.g., the occipital, sensorimotor, and Wernicke areas (27). They have also been recorded from the hippocampus and parahippocampal structures of healthy humans and they seem to play an important role in memory consolidation (28, 29). No parameter is able to perfectly distinguish pathological from physiological ripples (28, 30, 31).

Van 't Klooster et al. (26) demonstrated ioECoG recording from the mesiotemporal area in three patients with a temporal neocortical lesion, in whom only the lesion was resected. Two of them showed FRs in pre-ioECoG and they both had recurrent seizures; one patient did not show mesiotemporal FRs and was seizure-free (26). This finding, although based on small numbers, suggests that the presence or absence of FRs may have a good predictive power in lesional-free hippocampi of lateral neocortical patients with TLE.

Yu et al. (2021) recently studied the presence of spikes and HFOs in lesion-free hippocampi of neocortical patients with TLE and found no relation between events and seizure outcome (32). No other study has examined HFOs in detail in this specific cohort of patients.

We investigated the presence and the distribution of spikes and HFOs in patients with TLE, whose ipsilateral hippocampus is not affected by the lesion and is left *in situ* during surgery. We studied the events in the ioECoG before and after the resection and related their presence to the outcome. We aim to unravel whether spikes and HFOs can help us predict the postsurgical outcome in those patients with TLE whose mesial structures are not visibly affected by a lesion and who underwent hippocampus-sparing surgery.

## Methods

### Patients

We selected people who underwent ioECoG-tailored surgical resection of the temporal lobe in the UMC Utrecht between 2008 and 2017 from the RESPect database (Registry for Epilepsy Surgery Patients in the UMC Utrecht). Patients were included when:

- ioECoG was recorded at 2,048 Hz sampling frequency;
- post-ioECoG recordings were performed;
- photos of pre- and postresection grids placed on the cortex were available;
- the hippocampus was not involved in the lesion based on MRI and was left *in situ*;
- they were not included in ongoing clinical trials.

We excluded patients if they underwent chronic subdural ECoG monitoring or stereoelectroencephalography (sEEG) preceding surgery and if postsurgical seizure outcome after ≥1 year was not available.

The Medical Ethical Committee of the UMC Utrecht waived the need for informed consent for all retrospectively collected data before 2018 (as of 2018 an informed consent is required) and approved the use of coded data in the RESPect database for retrospective research (non-WMO; METC18-109C).

### Clinical Information

Baseline and clinical characteristics were collected, including gender, age at epilepsy onset, age at surgery, pathology findings, side of the resection (left/right), and postsurgical outcome.

We divided the pathology results into four subgroups: (a) low-grade and developmental epilepsy-associated tumors (LEAT); (b) focal cortical dysplasia (FCD); (c) others, such as cavernomas or encephaloclastic cyst, gliosis; (d) no abnormalities.

We collected the long-term postsurgical outcome, i.e., the most recent available follow-up after surgery with a minimum of 1 year. Postsurgical outcome was defined by the Engel classification, which we dichotomized into complete seizure freedom (Engel 1A) and recurrent seizures (Engel 1B-4).

### Intraoperative ECoG

The ioECoG was recorded by placing 4 × 5 or 8 × 4 grids on the cortex and in some cases a 1 × 6 or 1 × 8 strip in the subtemporal area (Ad-Tech, Racine, WI, USA). The grids and strips consist of platinum electrodes with 4.2 mm^2^ contact surface, embedded in silicone, placed with a 1-cm inter-electrode distance. Recordings were made with a 64-channel EEG system (Micromed, Treviso, Italy) at 2,048 Hz sampling rate with an antialiasing filter at 538 Hz.

Grids and strips were placed in multiple locations (“situations”) before and after the resection to cover the suspected epileptogenic tissue and the surrounding areas.

Every trace was recorded using conventional EEG settings (80 Hz low-pass filter, 10 seconds/page) in a common average reference montage.

The surgical plan was based on a comprehensive presurgical evaluation (always including Video-EEG, 3T MRI, neuropsychological testing, language testing with fTCD and/or fMRI, sometimes including PET, SPECT, EEG-fMRI, 7T MRI, WADA test) and discussed in our clinical multidisciplinary meeting, yielding the conclusion of the necessity of ioECoG-tailoring. The hippocampus-sparing approach was based on their amygdala and hippocampus appearing normal on MRIs (regardless of the distance from the lesion) and the other clinical parameters. The resection first included the whole pre-surgical MRI lesion irrespective of ioECoG findings, and additionally it was extended based on interictal spikes and ictiform spike patterns observed in the recording before the resection, called the pre-ioECoG. The ioECoG was repeated and the resection was extended when necessary. The recording after the final resection is called the post-ioECoG. HFOs were not analyzed during surgery in these patients. General anesthesia was induced and maintained with intravenous propofol, which is known to cause a burst-suppression pattern during ioECoG, inappropriate for surgical decision making. Therefore, propofol was interrupted during each ioECoG recording. An experienced anesthesiologist stopped propofol administration after ioECoG electrodes were in place, while analgesics and muscle relaxants were continued. Immediately after propofol interruption, a recording of about 10 min was done. Propofol administration was restarted directly after completion of each ioECoG recording or when evidence for light anesthesia arose (e.g., intraoperative movements, an increase in blood pressure or heart rate).

### Spike Analysis

From every recording, a 1-min epoch was selected for analysis. The chosen epoch was free from artifacts and close to the end of the recording to minimize the propofol effect. We excluded all channels with artifacts that did not allow proper marking from the analysis.

Spikes were visually marked by one author (AM) in BrainQuick Software (Micromed) and then checked by two other authors (MvtK and ES, when in doubt MZ or TG participated to help reach a consensus). The display was split vertically when marking spikes: on the left the reference montage used during the operation was shown and, on the right, a bipolar montage with conventional filter settings (80 Hz low-pass filter, IIR filter, gain of 600–1000 μV/cm, 10 s/page).

A spike was defined as a sharp transient, standing-out above baseline and lasting 80 ms maximum. Very sharp waves occurring at the same time on other bipolar channel traces were also marked as spikes.

Channels were marked as containing “ictiform spike patterns” when a continuous spiking, burst, and recruiting pattern was observed (26).

Some patients showed some burst-suppression (BS) in the selected minute despite the propofol stop. The BS signal in these patients affected <10% of the selected minute. In those cases, we visually marked bursts, defined as bursts of EEG activity clearly above the baseline and embedded in between suppression signal, and excluded the spikes and HFOs in these epochs from the analysis.

### HFO Analysis

High-frequency oscillations were visually marked by AM in BrainQuick and checked by two experts (MvtK/ES/MZ). Three time-synchronized screens were analyzed at the same time: one displayed the reference montage used during the operation; the second, where we marked ripples, showed a bipolar montage with a 80-Hz high-pass filter and a 50 μV/cm gain; the third, where we marked FRs, showed a bipolar montage with a 250 Hz high-pass filter and a 10 μV/cm gain. In both HFOs screens, we set a 1-s/page time window and an FIR filter. Only events containing at least four consecutive oscillations and clearly standing out above the background were marked as HFOs (33).

### ioECoG Pictures and Channels Annotation

Photos of the grids placed on the cortex were taken during pre- and post-ioECoG. Automated coregistration of these photos enabled us to schematize the electrode position in relation to the resection. Unipolar electrodes of the grids were classified as “resected”, “on edge” (≤0.5 cm from the resection margin), and “distant” (>0.5 cm from the resection margin). We excluded the unipolar electrodes placed on top of the resection cavity (post-ioECoG) from the analysis.

**Figure 1:**
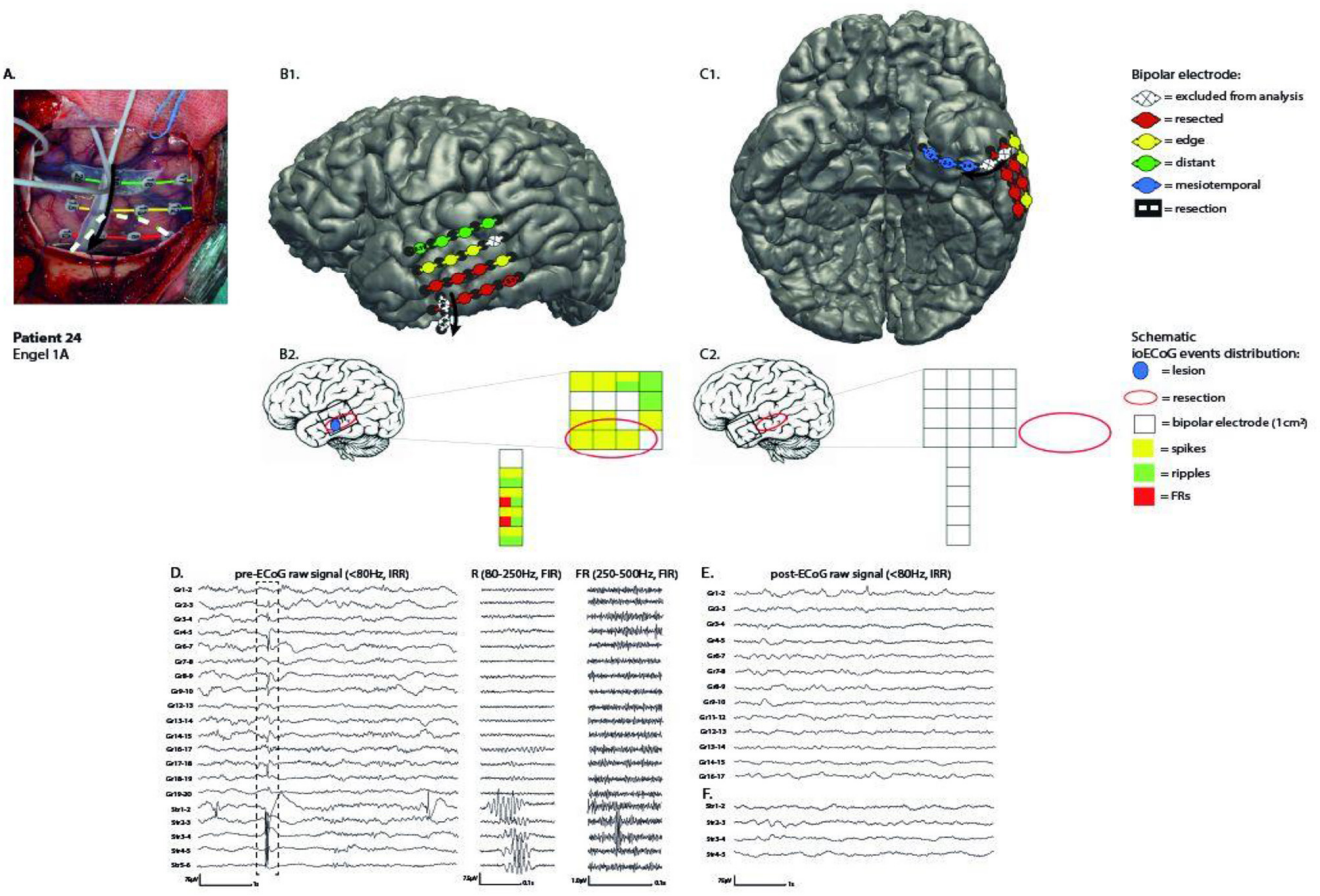
Example of patient 24, who underwent a left temporal lesionectomy to remove a cavernous hemangioma. Surgery was tailored with pre and post-ioECoG and the seizure outcome is good. **(A)** pre-ioECoG photograph. The area that will be furtherly resected is delineated by a white dotted line. Bipolar channels are highlighted with different colors based on the legenda on the right. **(B1)** Left hemisphere with a sketch of the neocortical grid. **(B2)** A schematic representation of pre-ioECoG grid and strip with the display of spikes and HFOs distribution. Each square represents a bipolar channel. **(C1)** Ventral view of the brain with a sketch of mesiotemporal strip. The three channels at the tip are the “mesiotemporal” ones and are highlighted in blue. **(C2)** A schematic representation of post-ioECoG grid and strip, where no event has been recorded. **(D)** Spikes and HFOs in pre-ioECoG. Note that HFOs are observed only on strip channels. **(E)** Grid post-ioECoG recording. **(F)** Strip post-ioECoG recording. Note that hippocampal spikes disappeared after the neocortical resection.

From this unipolar electrode classification, we derived the positions of bipolar channels as follows:

“Bipolar resected” when the two electrodes were both in the resected area, or one was resected and the other on edge;
“Bipolar on edge” when the two electrodes were on edge or one was on edge and the other distant;
“Bipolar distant” when the two electrodes were both distant (Figure 1).

These three channel categories refer to grid electrodes and display events recorded from the neocortex.

Strips were placed heading toward the mesiotemporal area and their tip was assumed to sample the mesiotemporal structures, e.g., hippocampus and entorhinal cortex. We classified the first three bipolar channels of the strips (Str1-2, 2-3, and 3-4) as “mesiotemporal,” representing a distinct channel category.

### Statistical Analysis

We split the events among the appropriate group of channels (“resected,” “edge,” “distant,” and “mesiotemporal”) for each patient. We calculated the mean event rate per minute over all channels with events (number of events/minute/channel with events).

We calculated the rates in “resected” channels (*RateRes)*, the rates in “edge” channels (*RateEdge*), the rates in distant channels (*RateDist*), the general rates on the whole neocortical channels (*RateNeo*), and the rates in “mesiotemporal” channels (*RateMesio*).

We calculated the rate resection ratio between pre-ioECoG events of “resected” channels and the pre-ioECoG events of the non-resected channels:

$$\begin{array}{l} \text{Rate resection ratio (ev)}=\frac{RateRes-\left(RateNonRes\;\right)}{RateAll} \end{array}$$

where *ev* is the type of the event, *RateRes* is the rate in “resected” channels, *RateNonRes* is the sum of *RateEdge, RateDist* and *RateMesio* when a mesiotemporal strip was placed. *RateAll* is the sum of rates in all channels.

Second, we performed a similar calculation based on the number of channels with events (#CwE) to evaluate the extent of resected channels regardless of the event rate.

$$\begin{array}{l} \text{Channel resection ratio (ev)}=\frac{\#CwERes-\left(\#CwENonRes\;\right)}{\#CwEAll} \end{array}$$

where *#CwERes* is the number of “resected” channels with events, *#CwENonRes* is the sum of the number of “edge” channels with events, “distant” channels with events, and “mesiotemporal” channels with events when a mesiotemporal strip was placed. *#CwEAll* is the sum of all channels with events. For both ratios, a value close to +1 indicates that most events/channels with events were resected, whereas a value close to −1 means that most of them were untouched.

We tested rates and resection ratios for differences between the recurrent-seizure and the seizure-free group. As our data were not normally distributed (Kolmogorov-Smirnov test), we used the Mann–Whitney *U* test for continuous variables. Categorical variables were compared using the χ^2^ test or Fisher exact test.

Last, a multiple logistic regression analysis was performed to investigate predictors of seizure freedom. We decided to select as covariates variables that could potentially influence both ioECoG findings and surgical outcome (demographic characteristics, **Table 2**) if their relatedness was confirmed by univariate analysis. We also selected *RateNeo* and *RateMesio* since they were our main objects of study. We selected *RateNeo* instead of *RateRes, RateEdge*, and *RateDist* in order to enhance the differences between the neocortex and mesiotemporal areas.

Since rates of events have an inter-dependency, we created a distinct logistic model per event type. Independent variables that reached statistical significance or showed a statistical trend were included in the final model. As an estimate of effect, we report adjusted odds ratios with 95% confidence intervals.

Since some patients did not have the mesiotemporal strip placed during ioECoG, we performed multiple imputations by chained equations with predictive mean matching to replace the missing values in the regression model.

Statistical analysis was performed in IBM SPSS Statistic 25 (IMB Corp., Armonk, NY). We considered *p* ≤ 0.05 significant.

## Results

### Patients

Between 2008 and 2017, 228 patients underwent ioECoG-tailored TLE surgery at the University Medical Center in Utrecht, the Netherlands. A total of 199 patients were excluded for various reasons: 68 had been already included in other ongoing clinical trials, 55 had ioECoG recorded at a sampling frequency lower than 2048 Hz, and 76 underwent a hippocampectomy. Out of the remaining patients, four were excluded since they had chronic ECoG recordings or SEEG before surgery, one patient was excluded as they were since lost to follow-up a few months after the operation and the known postsurgical seizure outcome was <1 year. This resulted in a total of 24 patients (Table 1).

**Table 1:** Patients and lesions characteristics.

**Patient #**	**Gender**	**Age at epi onset**	**Age at surgery**	**Surgery side**	**Pathology lesion**	**Lateral neocortical lesion location (MRI)**	**Engel class**
1	F	20	28	Right	Ganglioglioma WHO grade 1	Betweensuperior temporal gyrus and gyrus supramarginalis/gyrus angularis	1D
2	F	1	3	Left	Ganglioglioma WHO grade 1	Inferior temporal gyrus, anterior part	1B
3	M	4	7	Right	Encephaloclastic cyst	Anterior temporal lobe	1A
4	F	16	20	Right	Pleyomorphic xanthastocytoma	Between operculum temporalis and superior temporal gyrus	1A
5	M	23	28	Left	Ganglioglioma WHO grade 1	Inferior temporal gyrus	1A
6	M	3	13	Left	Oligoastrocytoma WHO grade 2	Basal temporal lobe	2A
7	M	10	19	Right	DNET WHO grade 1	Middle and posterior right temporal lobe	1A
8	F	34	40	Left	Oligoastrocytoma WHO grade 2	Basal temporal lobe	1A
9	M	3	4	Left	Tuberous sclerosis	Posterior temporal/anterior occipital lobe	2A
10	F	6	12	Left	DNET WHO grade 1	Anterior basal temporal lobe	1B
11	F	4	10	Right	FCD type 2B	Medial and superior temporal gyrus	1A
12	F	7	16	Right	Ganglioglioma WHO grade 1	Basal temporal lobe	1D
13	F	1	8	Right	FCD type 2A	Inferior temporal gyrus	2A
14	F	3	4	Left	Ganglioglioma WHO grade 1	Anterior temporal lobe, extending close to mesiotemporal areas	3A
15	M	11	12	Right	Ganglioglioma WHO grade 1	Superior temporal gyrus	1A
16	M	9	9	Right	DNET WHO grade 1	Temporal operculum	1A
17	M	13	16	Right	FCD Type 2B	Posterior temporal lobe	1A
18	F	11	16	Right	Ganglioglioma WHO grade 1	Lateral neocortex next to the anterior hippocampus	1A
19	M	12	17	Left	Ganglioglioma WHO grade 1	Inferior temporal gyrus, anterior part	1A
20	F	6	11	Right	FCD Type 2B	Superior temporal gyrus	1A
21	M	44	48	Left	No abnormality	MRI negative. PET shows hypometabolism in the entire left temporal lobe, except for mesiotemporal structures	1A
22	F	17	22	Right	FCD type 2B	Medial temporal gyrus	4A
23	M	13	13	Left	Ganglioglioma WHO grade 1	Inferior temporal gyrus	1B
24	M	35	39	Left	Cavernous hemangioma	Medial temporal gyrus	1A

### Surgery and Pathology

All patients were left-language dominant. Eleven patients underwent left-sided surgery and, among them, four were operated on while awake in a procedure incorporating language mapping. Pathology revealed tumors with a glial component in fifteen patients, FCD in five patients, other pathologies in three patients, and no abnormality in one patient (#21). In this last patient, MRI did not show any alteration, but PET showed hypometabolism in the whole left temporal lobe, except for mesiotemporal structures. Therefore, a left hippocampus-sparing anterior temporal lobectomy was performed. The postsurgery MRIs showed small residual tissue in four patients (#7, #8, #17, and #20) who all had good outcomes. In all the other 20 patients, no residual tissue was found. Patient characteristics can be found in Table 1.

### Postsurgical Outcome

The median (IQR) postoperative follow-up period was 39.0 (24.3–70.5) months. Ten out of 24 patients continued having seizures or relapsed. Nine out of those ten patients experienced a worthwhile improvement (Engel 1B-3C). Two patients (#14 and #22) were reoperated with a complete amygdalo-hippocampectomy, as already planned in case of poor outcome, unfortunately their outcome did not improve. The recurrent-seizure group had a significant younger age at seizure onset (U 48.50, *p* 0.01) (Table 2).

**Table 2:** Demographic characteristics of all patients.

	**Total**	**Seizure freedom**	**Recurrent seizures**	**Effect size**	***p***
		**Engel 1A**	**Engel 1B-4**		
No.	24	14	10		
M/F	12/12	9/5	3/7	OR: 0.28	0.21^a^
Age at epilepsy onset, y, median (IQR)	10.00 (4.0–16.0)	11.50 (8.25–25.75)	3.00 (2.00–10.00)	U: 48.50	0.01^*^^b^
Age at surgery, y, median (IQR)	14.50 (9.25–21.25)	16.50 (10.75–30.75)	12.50 (4.00–17.50)	U: 98.00	0.11^b^
Duration, y, median (IQR)	5.00 (2.00–6.00)	4.50 (3.00–5.25)	5.00 (1.00–8.00)	U: 59.00	0.83^b^
Follow-up period, mo, median (IQR)	39.00 (24.25–70.50)	33.50 (16.25–70.75)	56.00 (27.00–72.00)	U: 48.50	0.21^b^
Left/Right	11/13	5/9	6/4	OR: 2.70	0.41^a^
Pathology, % and *n*					1.00^a^
LEAT	15 (62,5%)	8	7		
FCD	5 (20,8%)	3	2		
Others	3 (12,5%)	2	1		
No abnormalities	1 (4,2%)	1	0		

*The last column shows the p-value for the difference between the good- and the poor-outcome group and the statistic test performed. We reported U and Odds Ratio (OR) as effect size. LEAT, Low-grade and developmental epilepsy associated tumors; FCD, Focal Cortical Displasia*.

a *Fisher exact test*.

b *Mann-Whitney U test*.

* *Significant (p ≤ 0.05)*.

### Pre-ioECoG

We included 996 bipolar channels in the pre-ioECoG analysis, with a median (IQR) of 36.5 (32.5–46.5) channels per patient. We excluded 182 bipolar channels because of artifacts, a median (IQR) of 6.0 (4.0–9.5) channels per patient. In total, 311 bipolar channels were classified as “resected”, 156 as “on edge,” and 421 as “distant”. In 17 patients, a sub-temporal strip was placed during pre-ioECoG. A total of 108 bipolar channels were considered “mesiotemporal.” One patient (#4) did not show any event.

All 23 other patients showed neocortical spikes, and among them, 6 showed ictiform spike patterns, 23 patients showed ripples, and 7 patients showed FRs on neocortical channels.

**Figure 2:**
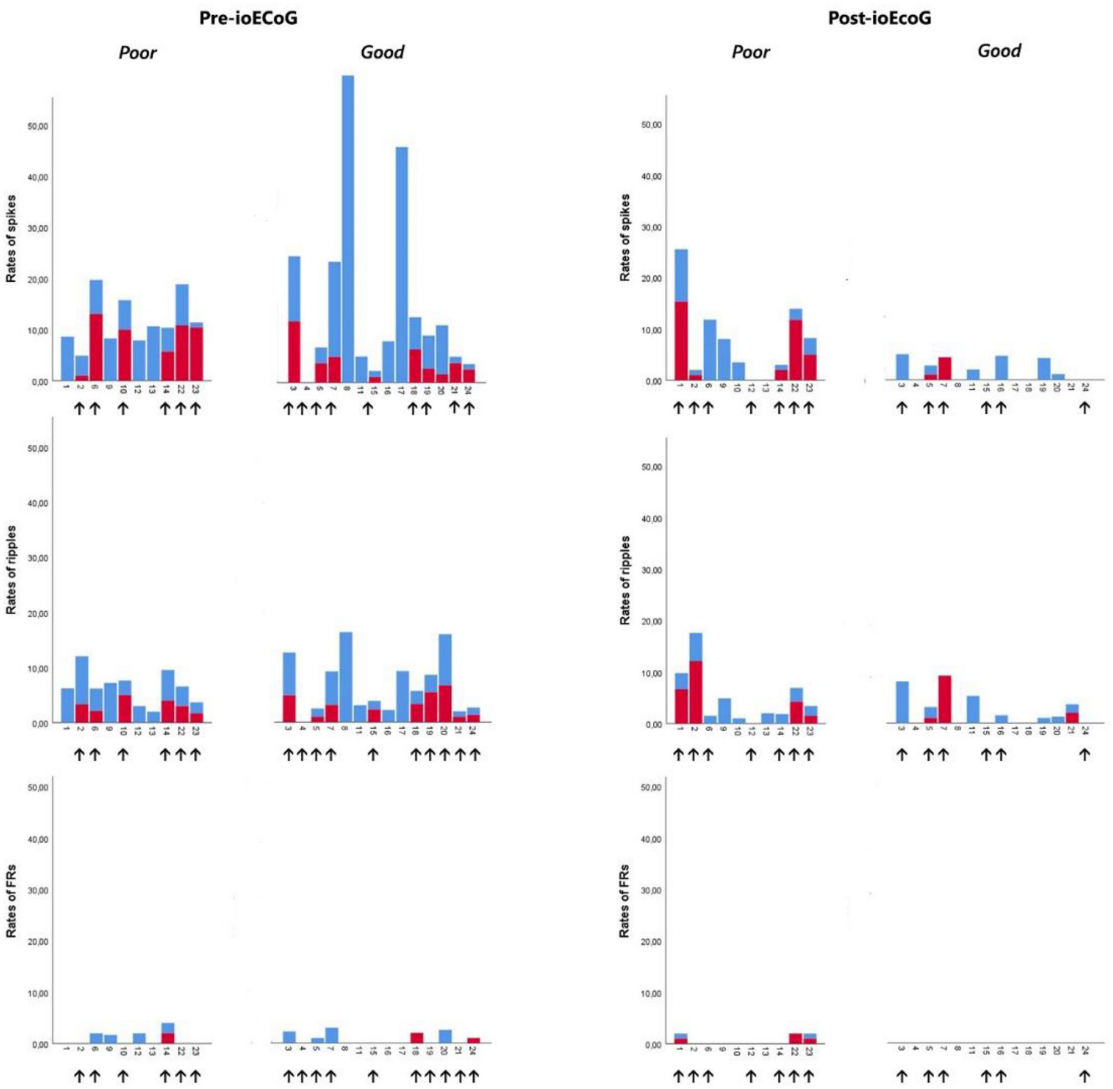
Pre- and post-ioECoG rates of spikes, ripples, and FRs in all patients over neocortical channels (light blue) and mesiotemporal channels (red). Patients are split based on the seizure outcome (“Poor” = Engel 1B-4; “Good” = Engel 1A). Black arrows indicate patients who had a mesiotemporal strip placed.

One patient showed no event on the mesiotemporal channels. All 16 other patients with a subtemporal strip showed spikes and ripples. In three patients, mesiotemporal FRs were recorded too. No ictiform spike patterns were observed on mesiotemporal channels (Figure 2).

### Post-ioECoG

We included 528 bipolar channels in the post-ioECoG analysis, with a median (IQR) of 17.5 (13.7–28.2) channels per patient. We excluded 186 bipolar channels because of artifacts or position above the cavity, a median (IQR) of 4.5 (3.2–11.2) channels per patient. In 13 patients, a subtemporal strip was placed during post-ioECoG. In total 100 bipolar channels were classified as “on edge,” 372 as “distant,” and 56 were classified as “mesiotemporal.

Six patients did not show any residual events.

One patient showed an ictiform spike pattern and 14 patients showed residual spikes on neocortical channels. Ripples were recorded in 16 patients, FRs were observed on neocortical channels in two patients.

In four patients, no event was found on the strip electrodes over the mesiotemporal areas. Spikes were marked on mesiotemporal channels in 7 out of the 13 patients with a subtemporal strip. Mesiotemporal ripples were found in eight patients. Three patients showed FRs on the post-ioECoG strip. No ictiform spike patterns were observed on mesiotemporal channels (Figure 2).

### Descriptive Comparison of Pre- and Post-ioECoG and Relation to Outcome

One patient did not show any events before and after the resection and belongs to the seizure-free group. Twenty-three patients had spikes and ripples in pre-ioECoG. Six of them did not have any residual event in the post-ioECoG: five were seizure-free and one, in whom the mesiotemporal structures were not sampled with post-ioECoG, still had seizures.

The ictiform spike patterns were all on resected channels except in one patient with a poor outcome, who displayed them on the lateral neocortex in both the pre- and post-ioECoG. The surgeon could not extend the resection as it would affect eloquent areas.

Out of the eight patients with neocortical FRs in the pre-ioECoG, none had residual FRs in the post-ioECoG, and three of them belonged to the poor-outcome group. Two patients with neocortical FRs in the post-ioECoG did not show FRs before the resection and they both had a poor outcome.

Two out of three patients with mesiotemporal FRs in the pre-ioECoG did not show them in the post-ioECoG strip after the neocortical resection. One is seizure-free, one had recurrent seizures and underwent a complete amygdalo-hippocampectomy 3 months after the first surgery, but it did not show an improved outcome. The third patient was seizure-free, but the strip was not placed during post-ioECoG.

Three patients showed FRs on the post-ioECoG strip and they all belonged to the poor-outcome group*:* in two of them no FR was marked over the mesiotemporal areas before the resection. In one of them, a complete amygdalo-hippocampectomy was performed 2 months after the first surgery, but the outcome did not improve. In the third one, the strip was not placed during pre-ioECoG.

### Statistical Comparison to Seizure Outcome

#### Neocortex

In pre-ioECoG, *RateRes* and *RateEdge* of all events were similar between the two outcome groups. *RateDist* of spikes and FRs did not show differences, while *RateDist* of ripples were higher in the recurrent seizure than in the seizure-free group (U 22.50, *p* 0.04; Table 3).

**Table 3:** Median (IQR) of pre and post-ioECoG rates for all channel categories in the good- and poor-outcome groups.

		**Resected**				**Edge**				**Distant**				**Mesiotemporal**			
		**Median (IQR) Engel 1A**	**Median (IQR) Engel 1B-4**	***U***	***p***	**Median (IQR) Engel 1A**	**Median (IQR) Engel 1B-4**	***U***	***p***	**Median (IQR) Engel 1A**	**Median (IQR) Engel 1B-4**	***U***	***p***	**Median (IQR) Engel 1A**	**Median (IQR) Engel 1B-4**	***U***	***p***
**Pre-ioEcoG**	Spikes	7.18 (1.00–19.61)	8.08 (1.53–9.88)	73.00	0.89	3.10 (1.00–9.62)	3.16 (0.99–7.62)	75.00	0.80	1.64 (0.00–4.69)	4.00 (1.58–8.08)	44.00	0.14	3.00 (1.37–7.10)	4.67 (1.00–11.09)	28.50	0.54
	Ripples	2.33 (0.00–10.72)	2.08 (0.00–7.72)	80.00	0.58	2.50 (0.75–8.31)	2.80 (1.00–6.16)	70.00	1.00	1.76 (0.75–2.67)	3.79 (2.60–5.80)	22.50	0.04^*^	2.67 (1.25–4.37)	3.17 (1.00–4.90)	35.50	1.00
	FRs	0.00 (0.00–0.25)	0.00 (0.00–1.25)	60.00	0.58	0.00 (0.00–0.37)	0.00 (0.00–0.25)	72.00	0.93	0.00 (0.00–0.00)	0.00 (0.00–0.50)	67.00	0.89	0.50 (0.00–1.25)	0.00 (0.00–0.00)	45.50	0.31
**Post-ioEcoG**	Spikes					0.00 (0.00–1.00)	1.50 (0.00–4.00)	41.00	0.10	0.00 (0.00–2.15)	2.16 (1.00–9.00)	36.50	0.05^*^	0.00 (0.00–5.00)	1.00 (0.00–7.13)	18.00	0.73
	Ripples					0.00 (0.00–0.25)	1.50 (0.00–1.80)	33.50	0.03^*^	0.50 (0.00–1.78)	2.12 (1.37–4.16)	36.50	0.05^*^	0.00 (0.00–2.00)	3.83 (0.00–9.98)	13.00	0.29
	FRs					0.00 (0.00–0.00)	0.00 (0.00–0.00)	70.00	1.00	0.00 (0.00–0.00)	0.00 (0.00–0.25)	62.00	0.67	0.00 (0.00–1.00)	0.00 (0.00–0.25)	24.00	0.73

*We tested for differences between the two outcome groups with Mann Whitney U test. U and p-values are displayed*.

* *Significant (p ≤ 0.05)*.

In post-ioECoG, *RateEdge* of spikes and FRs were not different between the two outcome groups, while *RateEdge* of ripples were higher in patients with poor outcome than in patients with good outcome (U 33.50, *p* 0.03). The recurrent-seizure group showed higher *RateDist* of spikes and ripples than the seizure-free group (spikes: U 36.50, *p* 0.05; ripples: U 36.50, *p* 0.05), but no difference in the FRs rates (Table 3).

#### Mesiotemporal Areas

In pre-ioECoG, *RatesMesio* of all events did not differ between patients with good and poor outcome. The same was true for post-ioECoG (Table 3).

#### Resection Ratios

**Figure 3:**
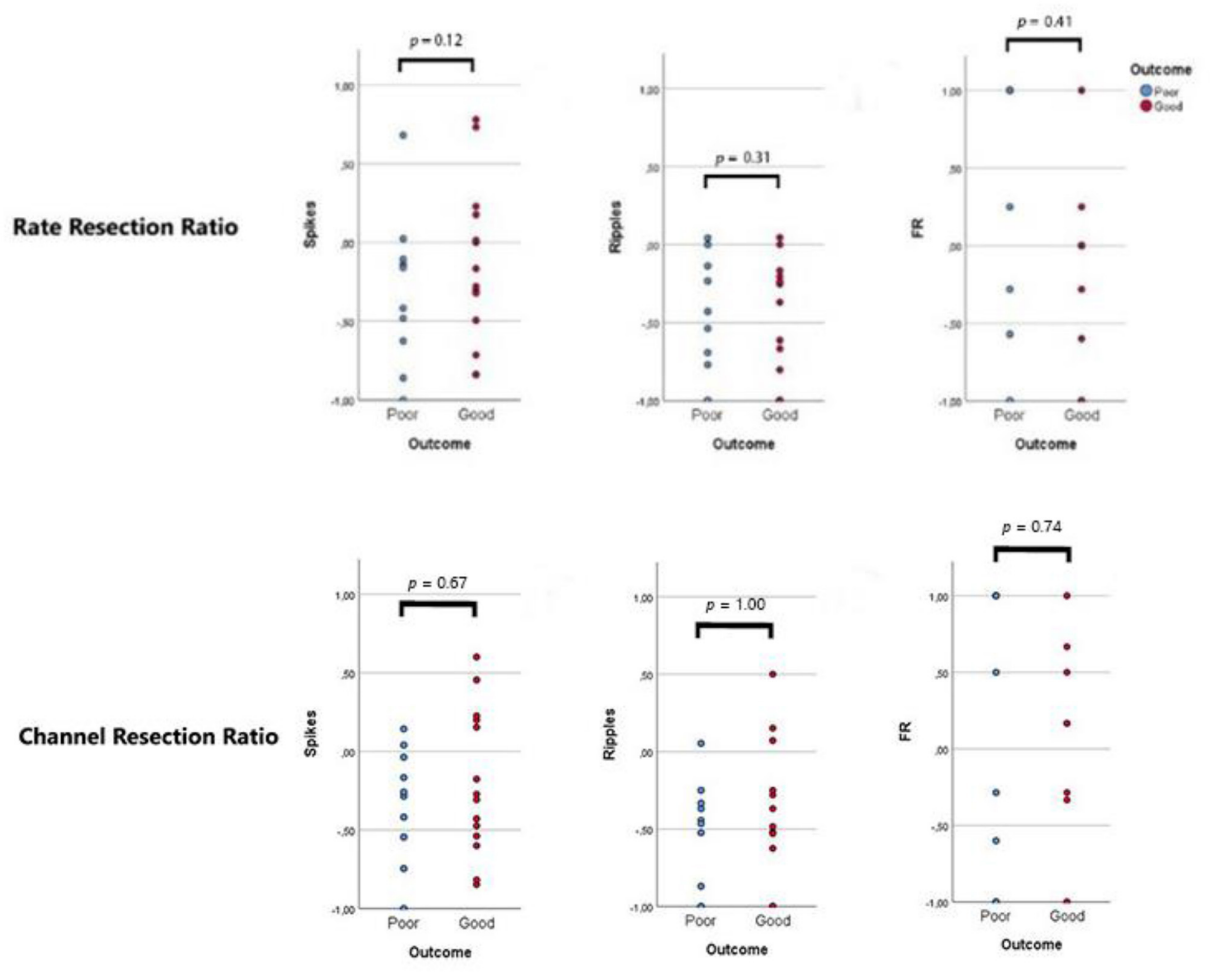
Resection ratios. Each dot represents a patient: blue dots refer to patients with recurrent seizures (“poor”, Engel 1B-4), red dots indicate seizure-free patients (“good”, Engel 1A). A ratio >0 indicates that the majority of events/channels with events were resected during surgery. No difference was observed between the two outcome groups.

Rate resection ratios and channel resection ratios did not display differences between good- and poor-outcome patients (Figure 3).

#### Multiple Logistic Regression

Besides *RateNeo* and RateMesio, *age at epilepsy onset* was selected as covariate to the multiple logistic regression model because of the potential relation with ioECoG findings (34) and of the result of the univariate analysis. No significant predictors of seizure outcome were found in the spikes- and the FRs-based-model. In the ripple-based model, older age at epilepsy onset (OR 1.76, 95%CI 1.03–3.02, *p* 0.04) and low rates of mesiotemporal events in post-ioECoG (OR 0.13, 95%CI 0.20–0.88, *p* 0.04) were significant predictors of good seizure outcome (Table 4). The sensitivity of this model was 78% and its specificity 80%.

**Table 4:** Multiple logistic regression analysis of potential predictors of seizure freedom (*n* = 24).

**RIPPLES**	**Variables**	**B**	**S.E**.	**OR**	**95% CI for OR**		***p-*value**
					**Low**	**Upper**	
	Age at epilepsy onset	0.57	0.27	1.76	1.03	3.02	0.04^*^
	*RateNeo*	1.30	0.68	3.68	0.97	13.9	0.06
	*RateMesio*	−2.02	0.96	0.13	0.02	0.88	0.04^*^
	Constant	−5.46	2.79	0.004			0.05^*^

*The analysis here displayed is based on rates of ripples and shows only the independent variables that reached statistical significance or a trend. Parameters (B), standard errors (S.E.), odds ratios(OR) and 95% confidence intervals are presented*.

*General statistics about the model:−2 Log Likelihood: 17.16; R2 = 0.48 (Cox and Snell), 0.64 (Nagelkerke); Model χ^2^ = 15.45; model p-value: < 0.01*.

* *Significant p-value ≤ 0.05*.

## Discussion

The ioECoG of poor-outcome patients showed higher rates of pre-resection ripples and residual spikes and ripples in channels distant from the resection outline. Rates of mesiotemporal events were similar among the two outcome groups. Residual FRs and ictiform spike patterns were found only in poor-outcome patients. In our cohort, low rates of mesiotemporal post-ioECoG ripples and older age at epilepsy onset were significant predictors of good seizure outcome. High rates of lateral neocortical ripples in post-ioECoG were a trend predictor (*p* = 0.06) of good seizure outcome. To conclude, HFOs may help inform the extent of successful surgical resection and chances of favorable outcome following ioECoG-guided resection in lateral neocortical temporal epilepsy.

In the Mann–Whitney U tests, higher rates of residual ripples on the neocortex on edge and distant channels were linked to poor outcome, while in the logistic regression model a higher rate of ripples over the neocortex seemed to predict good outcome. To explain these seeming contradictions, we must realize that both epileptic and physiological ripples may be age-dependent. A previous study showed that ripples frequency significantly decreases with age in relation to brain maturation and regardless of the seizure control (34): in other words, the younger the patient (at epilepsy onset), the more ripples may be found. Age of epilepsy onset is thus related both to ripple rates and seizure outcome. The regression model provides therefore a different relation between neocortical ripples and seizure outcome when adjusted for age of onset.

Besides, although we were not able to distinguish physiological from pathological ripples, we hypothesized that the presence of physiological ripples could have impaired our analysis and led to these challenging results. The coupling of ripples with epileptic spikes has been studied as a marker that was able to discriminate pathological from physiological ripples (28, 31). We had a too small of a cohort to perform meaningful analysis on a multitude of variables such as HFOs co-occurrence with or without spikes. We had a look to the side of TLE as a confounder, since theoretically the dominant hemisphere (in all of our patients it was the left) should include more eloquent areas and show a higher rate of physiological HFOs. No clear difference was observed between left- and right-sided TLE groups, but it is suggested to investigate this issue in future studies.

We took inspiration from the study of Jacobs et al. (2010) (16) for the formulas of rate and channel resection ratios. In their study, the rate resection ratio was significantly higher in good-outcome patients, while in our study the percentage of events/channel with events included in the resected areas was not relevant in comparison to the presence of high rates of residual events. We included in the “non-resected” events and channels also the mesiotemporal ones, and this could partly explain the difference in the results of the two studies.

Our study showed different results from Yu et al. (2021) (32), who observed a good outcome in 90% of patients who underwent a lesionectomy in TLE with intact mesial structures and found that ioECoG did not add any useful information. In our study, only the 67% of our cohort had good outcome after the neocortical temporal resection, and post-ioECoG was useful to predict postsurgical seizure outcome and to possibly lead the neurosurgical team to extend the resection when necessary. The difference between the Korean results and ours partly depends on the different categorization of outcome groups: Yu et al. (32) included in the seizure-free group patients belonging to Engel 1A-1B, while we included the Engel 1As only. Also, we performed intraoperative ECoG in patients in whom we did not doubt the surgical plan and thus we anticipated a good outcome.

Our original study idea was to investigate if spikes and HFOs could help discriminate which patients with NTLE would benefit from an amygdalo-hippocampectomy, but the number of patients who underwent a secondary hippocampectomy proved too small for proper analysis (only two patients); thus we focused on the prediction of success chance in our cohort. During the first surgeries of these two patients (#14 and #22), ioECoG recorded many events in their mesiotemporal structures both before and after the resection (FRs too). The neurosurgical team decided to leave those structures *in situ* and to reevaluate the condition in a few months. Seizures were still very frequent and #14, in particular, had a seizure semiology compatible with mesiotemporal epilepsy. The post-MRI showed left hippocampal atrophy not observed in the MRIs before the first operation and an EEG recorded the seizures from the left temporal lobe. Subject #22's post-MRI found gliosis surrounding the cavity resulting from the first operation and intact mesiotemporal areas. It was decided to reoperate those two patients, and during the second surgeries, pre-ioECoG grids and strips recorded clear mesiotemporal epileptic activity. A resection extension plus an amygdalo-hippocampectomy was performed in both patients, but the outcome did not improve in either one of them. These two reoperated patients demonstrate that the resection of mesiotemporal areas showing seeming epileptic events does not always improve seizure outcome. We hypothesized that maybe both mesiotemporal lobes of the patients were involved in the epileptogenic network and that the homolateral amygdalohippocampectomy was not sufficient to decrease seizure frequency.

A positive aspect of our study is that we included a very specific and unique population that underwent the same presurgical and intraoperative workflow, avoiding many possible biases. On the other side, this strict selection led to a low number of patients studied. The mesiotemporal strip was not placed in all patients, thus we had some missing information that we handled with multiple imputation. For both research, as well as clinical purpose, it is important to standardize the ioECoG-tailoring approach of patients with TLE, placing a mesiotemporal strip even if amygdala and hippocampus are not involved in the lesion according to the MRI.

A limitation of our study is that our ioECoG recordings are affected by artifacts generated by surgical equipment: in particular, FRs marking was impaired by the presence of high noise in some patients. We became aware that the 64 channels LTM express headbox (Micromed, Treviso), which was used in 14 of our patients, produces more noise in the FR band than other headboxes. This highlights the importance of the use and development of the right equipment, e.g,. that reduces noise as much as possible for a proper ioECoG recording and subsequent HFOs marking. The recordings were also affected by end-stage burst suppression due to propofol anesthesia. HFOs increase after stopping propofol, but we do not know how this relates to the occurrence of physiological and pathological events. An analysis focused on HFOs occurring during BS could be of great interest for the future of ioECoG interpretation and its clinical use.

IoECoG has a limited spatial sampling. We included in our cohort only patients who had not been investigated with chronic ECoG recording before surgery, thus we did not sample either the contralateral temporal lobe or other areas of the homolateral hemisphere. Therefore, it is possible that poor-outcome patients might have had some distant epileptogenic focus that was not recorded during ioECoG. For mesiotemporal areas, direct sampling with depth electrodes may have been more accurate. We also perform sEEG evaluation in our center, but we use ioECoG in case of suspected unilateral epilepsy with a single focus, and we commonly use strip electrodes to sample mesiotemporal structures during these intra-operative recordings. When placed correctly, a strip electrode directed toward the mesio-temporal structures shows distinct spike-activity in case the hippocampus is affected and in addition allows for monitoring of involvement of the basal-lateral neocortical tissue.

In conclusion, this study shows more evidence of the prognostic role of post-ioECoG for seizure outcome in lateral neocortical temporal epilepsy. To address how to discriminate patients with NTLE who will benefit from an amygdalo-hippocampectomy, a prospective study, including a control group without ioECoG would be required. A more extensive prediction model of ripples and FRs in the postresection mesiotemporal structures should also take clinical parameters like neuropsychological testing and MRI results into account.

## Data Availability Statement

The raw data supporting the conclusions of this article will be made available by the authors, without undue reservation.

## Ethics Statement

The studies involving human participants were reviewed and approved by Medical Ethical Committee of UMC Utrecht METC18-109C. Written informed consent from the participants' legal guardian/next of kin was not required to participate in this study in accordance with the national legislation and the institutional requirements.

## RESPect Database Study Group

Angelika Muhlebner, Anouk Velders, Charlotte van Asch, Cyrille Ferrier, Dora Hermes, Dorien van Blooijs, Floor Jansen, Dongqing Sun, Eline Schaft, Eva Zuidhoek, Frans Leijten, Friso Hoefnagels, Geertjan Huiskamp, Gerard de Kort, Harriët Smeding, Jack Zwemmer, Jan-Willem Dankbaar, Janine Ophorst, Kees Braun, Lydia van den Berg, Maeike Zijlmans, Mariska Mantione, Martine van Zandvoort, Maryse van 't Klooster, Matteo Demuru, Merel Wassenaar, Mireille Bourez, Monique Hobbelink, Monique Schooneveld, Nicole van Klink, Paul Smits, Peter Gosselaar, Peter van Rijen, Pierre Robe, Pieter van Eijsden, Renate van Regteren, Sifra Blok, Sandra van der Salm, Tineke Gebbink, Willemiek Zweiphenning.

## Author Contributions

AM, ES, MvtK, and MZ contributed to study concept, design, data analysis, and manuscript preparation. WZ, MD, PG, TG, and WO contributed to the acquisition of data, to the review of content and statistical analysis, and to the interpretation of the results. All authors approved the submitted version.

## Funding

MZ, ES, and MvtK were supported by ERC Starting Grant 803880. MvtK is co-funded by the PPP Allowance made available by Health Holland, Top Sector Life Sciences & Health.

## Conflict of Interest

The authors declare that the research was conducted in the absence of any commercial or financial relationships that could be construed as a potential conflict of interest.

## Publisher's Note

All claims expressed in this article are solely those of the authors and do not necessarily represent those of their affiliated organizations, or those of the publisher, the editors and the reviewers. Any product that may be evaluated in this article, or claim that may be made by its manufacturer, is not guaranteed or endorsed by the publisher.
