# Supplementary material for: Spikes and High Frequency Oscillations in Lateral Neocortical Temporal Lobe Epilepsy: Can They Predict the Success Chance of Hippocampus-Sparing Resections?
Source: Front Neurol. 2022 Aug 1;13:797075. doi: 10.3389/fneur.2022.797075 (PMC9379925; doi:10.3389/fneur.2022.797075)

*
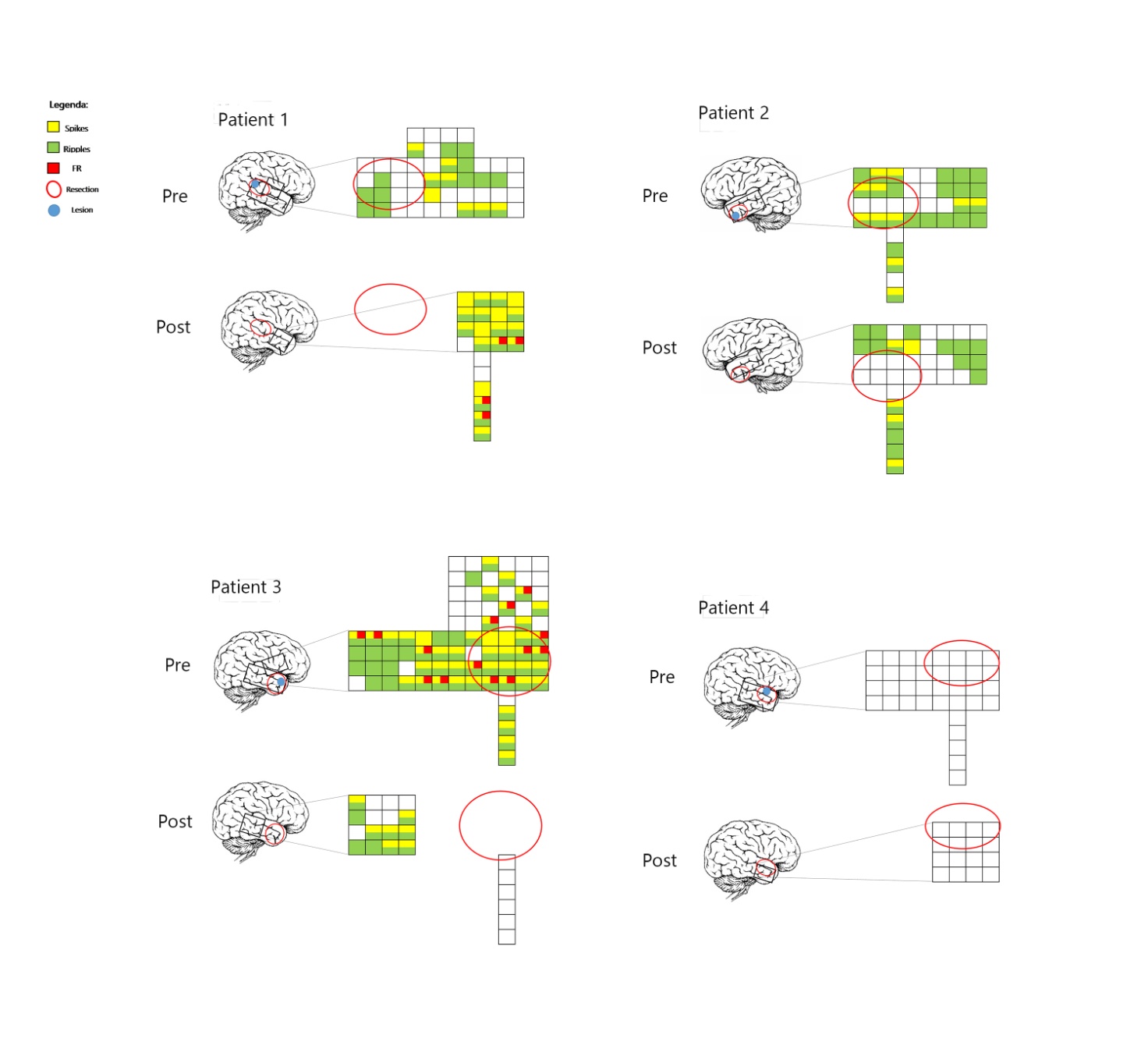
Figure 1*. Schematic representation of ioECoG events distribution. For every patient, we depicted the proper hemisphere, the approximate location of the grid, the resection and the lesion. The ioECoG grids and strip are zoomed in a sketch where each square represents a bipolar channel and is coloured based on the events recorded (see *Legenda*).


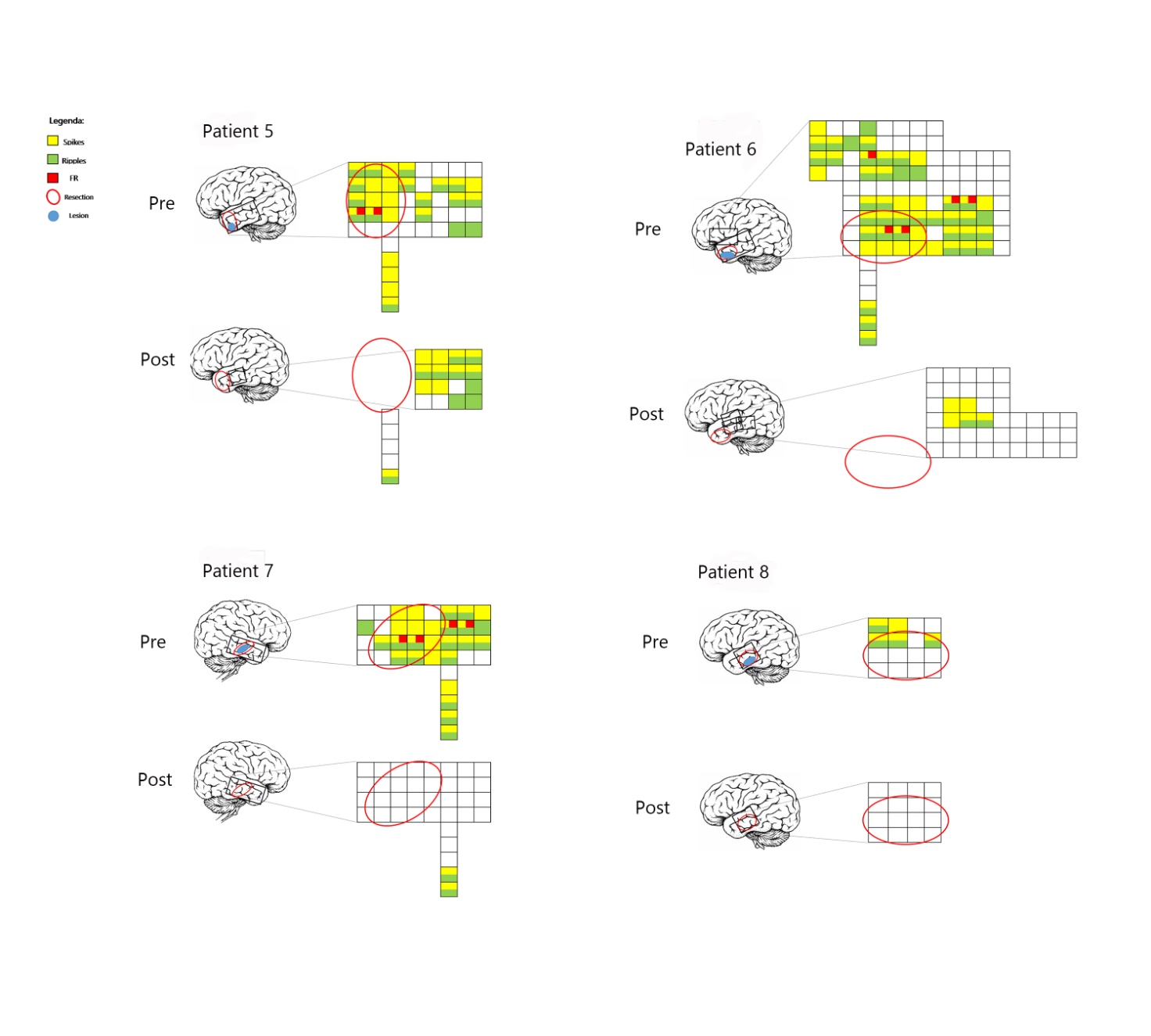


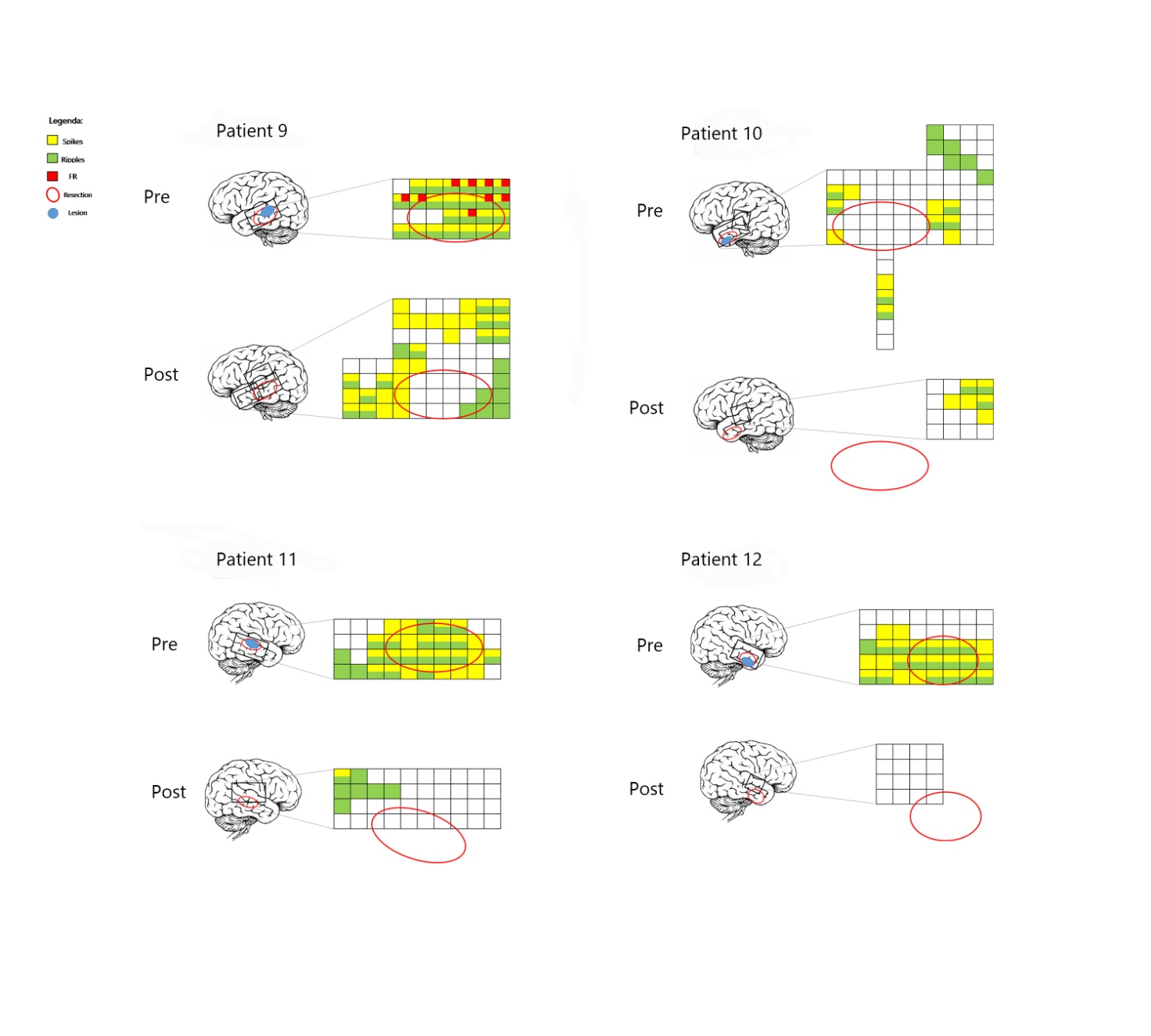


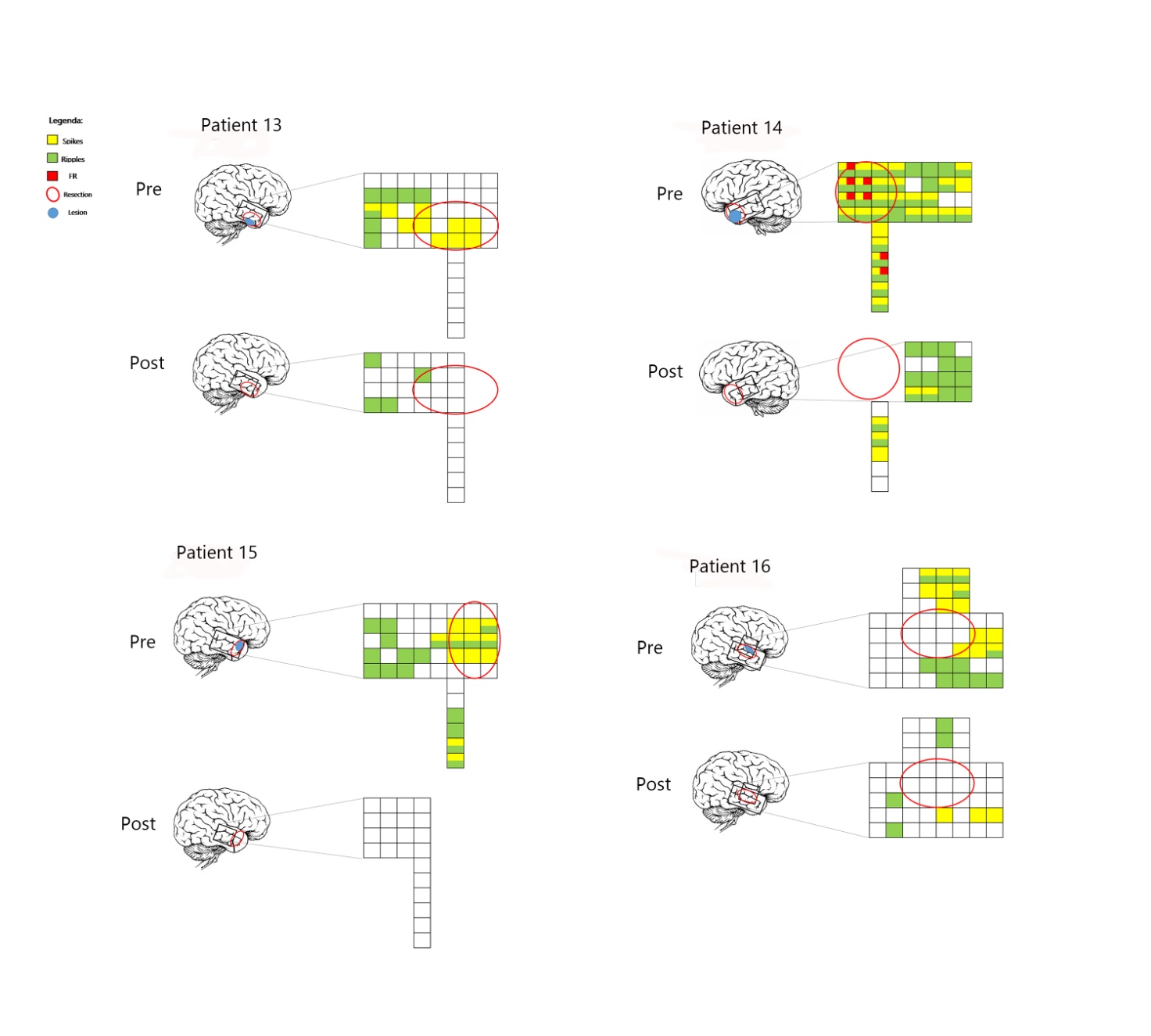


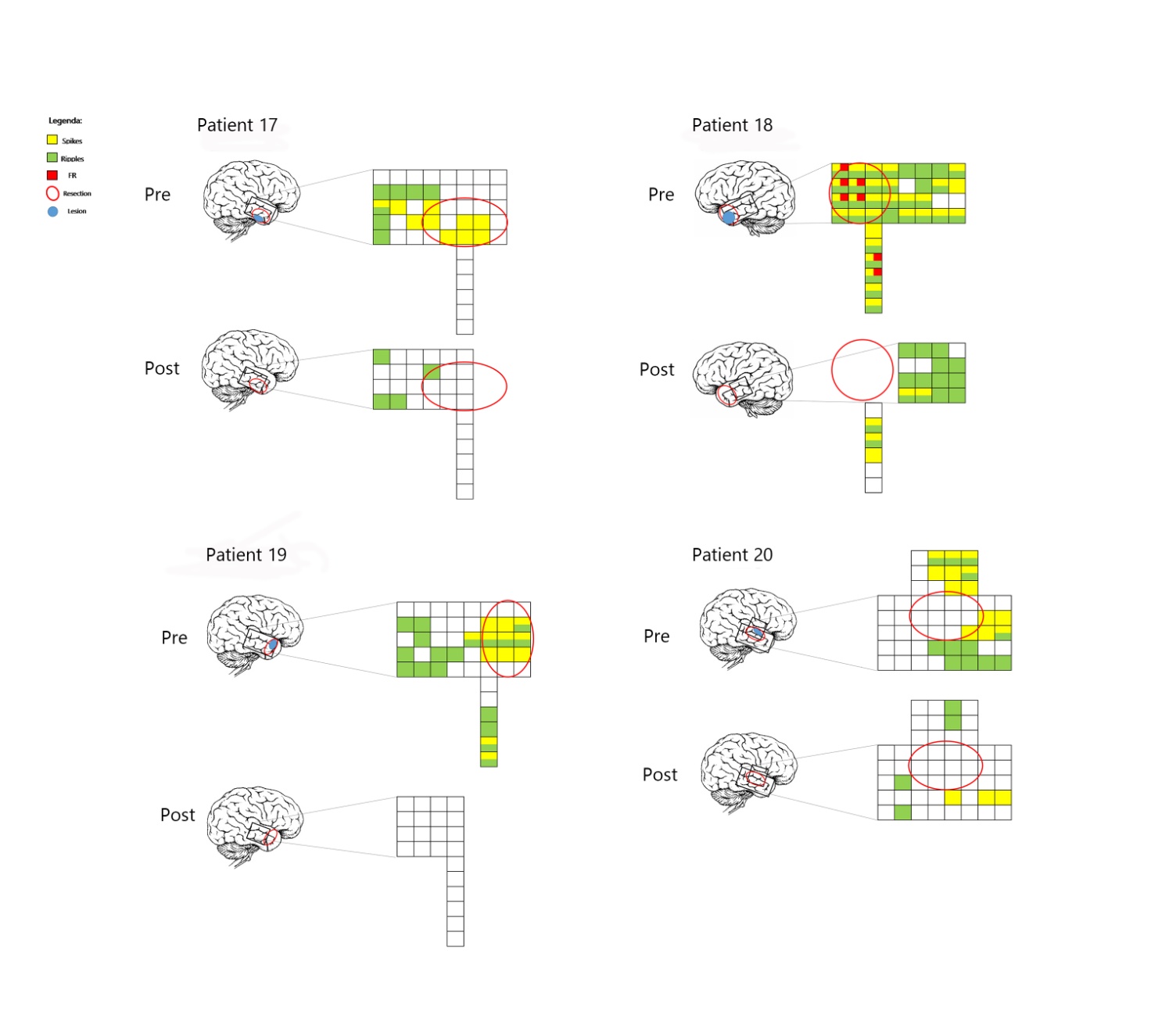


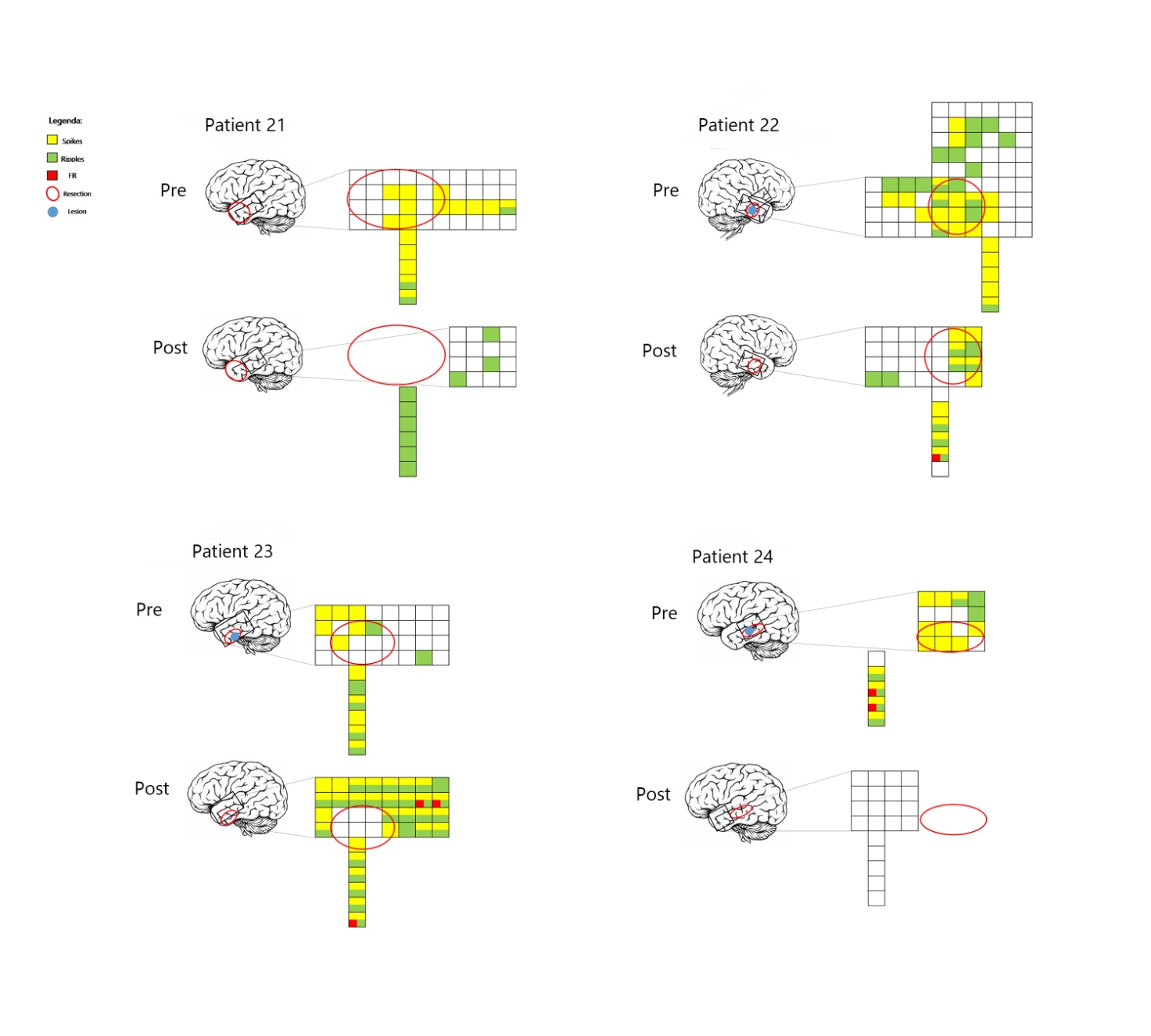

Supplement: Supplementary file 1 [file Data_Sheet_1.docx]
